# Nutritional treatment of children 6–59 months with severely low weight-for-age *z*-score: a study protocol for a 3-arm randomized controlled trial

**DOI:** 10.1186/s13063-023-07890-0

**Published:** 2024-01-08

**Authors:** Suvi T. Kangas, Césaire T. Ouédraogo, Moctar Tounkara, Bareye Ouoluoguem, Issa Niamanto Coulibaly, Alhousseyni Haidara, Niele Hawa Diarra, Koniba Diassana, Zachary Tausanovitch, Christian Ritz, Jonathan C. Wells, André Briend, Mark Myatt, Elizabeth Radin, Jeanette Bailey

**Affiliations:** 1 International Rescue Committee, New York, USA; 2 International Rescue Committee, Bamako, Mali; 3 Department of Education and Research in Public Health and Specialties, Faculty of Medicine and Dentistry, University of Sciences, Technics and Technologies of Bamako, Bamako, Mali; 4 Sub-Direction of Nutrition, Ministry of Health, Bamako, Mali; 5 National Institute of Public Health, Copenhagen, Denmark; 6 Population Policy and Practice Department, UCL Great Ormond Street Institute of Child Health, London, UK; 7 Department of International Health, University of Tampere School of Medicine, Tampere, Finland; 8 Department of Nutrition, Exercise and Sports, Faculty of Sciences, University of Copenhagen, Copenhagen, Denmark; 9 Brixton Health, Brixton, UK; 10 Emergency Nutrition Network, Kidlington, Oxforshire, UK

**Keywords:** Acute malnutrition, Community-based management of acute malnutrition, Efficacy, Mali, Mid-upper-arm circumference, Moderate acute malnutrition, Ready-to-use therapeutic food, Relapse, Severe acute malnutrition, Simplified protocol, Stunting, Treatment, Weight-for-age *z*-score, Underweight, Wasting

## Abstract

**Background:**

Admission criteria that treat children with low mid-upper-arm circumference (MUAC), and low weight-for-height *z*-score (WHZ) are not aligned with the evidence on which children are at risk of mortality. An analysis of community-based cohort data from Senegal found that a combination of weight-for-age (WAZ) and MUAC criteria identified all children at risk of near-term death associated with severe anthropometric deficits. This study will address whether children with WAZ <−3 but MUAC ≥125 mm benefit from therapeutic feeding with ready-to-use therapeutic foods (RUTF) and whether a simplified protocol is non-inferior to the weight-based standard protocol.

**Methods:**

This is a prospective individually randomized controlled 3-arm trial conducted in the Nara health district in Mali. Children aged 6–59 months presenting with MUAC ≥125 mm and WAZ <−3 will be randomized to (1) control group receiving no treatment, (2) simplified treatment receiving 1 sachet of RUTF daily until WAZ ≥−3 for 2 visits, (3) standard treatment receiving RUTF according to WHZ category: (a) WHZ <−3 receive 200 kcal/kg/day until WHZ ≥−2 for 2 visits, (b) WHZ ≥−3 but <−2 receive 1 sachet daily until WHZ ≥−2 for 2 visits or (c) WHZ ≥−2 receive no treatment. All children will be followed up first fortnightly for 12 weeks and then monthly until 6 months post-enrolment. The primary endpoint will be measured at 2 months with the primary outcome being WAZ as a continuous measure. Other outcomes include other anthropometric measurements and a secondary endpoint will be observed at 6 months. A total of 1397 children will be recruited including 209 in the control and 594 in both the simplified and standard arms. The sample size should enable us to conclude on the superiority of the simplified treatment compared to no treatment and on the non-inferiority of the simplified treatment versus standard treatment with a margin of non-inferiority of 0.2 WAZ.

**Discussion:**

This trial aims to generate new evidence on the benefit of treating children with WAZ <−3 but MUAC ≥125 mm in order to guide the choice of admission criteria to malnutrition treatment and build evidence on the most efficient treatment protocol.

**Trial registration:**

This trial was registered at ClinicalTrials.gov: NCT05248516 on February 21, 2022.

**Supplementary Information:**

The online version contains supplementary material available at 10.1186/s13063-023-07890-0.

## Administrative information

Note: the numbers in curly brackets in this protocol refer to SPIRIT checklist item numbers. The order of the items has been modified to group similar items (see http://www.equator-network.org/reporting-guidelines/spirit-2013-statement-defining-standard-protocol-items-for-clinical-trials/). The SPIRIT check-list document can be found in supplementary information.

**Table:** 

Title {1}	Nutritional treatment of children 6–59 months with low weight-for-age *z*-score: a study protocol for a 3-arm individually randomized controlled trial
Trial registration {2a and 2b}.	This trial was registered at ClinicalTrials.gov: NCT05248516.
Protocol version {3}	Version 3.2, November 15^th^ 2022.
Funding {4}	Thompson Foundation and Sheryl Sandberg & Tom Bernthal Family Foundation.
Author details {5a}	1 International Rescue Committee, New York, USA 2 International Rescue Committee, Bamako, Mali 3 Department of Education and Research in Public Health and Specialties, Faculty of Medicine and Dentistry, University of Sciences, Technics and Technologies of Bamako, Bamako, Mali 4 Sub-Direction of Nutrition, Ministry of Health, Bamako, Mali 5 National Institute of Public Health, Copenhagen, Denmark 6 Population Policy and Practice Department, UCL Great Ormond Street Institute of Child Health, London, UK 7 Department of International Health, University of Tampere School of Medicine, Tampere, Finland 8 Department of Nutrition, Exercise and Sports, Faculty of Sciences, University of Copenhagen, Copenhagen, Denmark 9 Brixton Health, UK 10 Emergency Nutrition Network, Kidlington, Oxforshire, UK
Name and contact information for the trial sponsor {5b}	Jeanette Bailey Director of Nutrition Research and Innovation Airbel Impact Lab International Rescue Committee Jeanette.bailey@rescue.org 122 E. 42nd St, New York, NY, USA 10168
Role of sponsor {5c}	The study sponsor, IRC, was responsible for developing the study idea, obtaining funding for the study, designing the study, implementing it and analyzing and interpreting study results. Funders were not involved in developing the study design, methods, implementation or data analysis.

## Introduction

### Background and rationale {6a}

The field of nutrition divides undernutrition into two categories: micronutrient deficiencies and growth deficits. Micronutrient deficiencies arise when specific type 1 micronutrients, such as iron, are lacking in the diet [1]. These deficits give rise to a specific symptom, anemia in the case of iron, that is rather easy to identify [2] and treat via supplementation [3, 4]. Type 1 micronutrients include all vitamins as well as some minerals such as iodine, copper, and calcium [1]. Growth deficits occur when either type 2 micronutrients or macronutrients are lacking in the diet leading to slowed height and weight gain. Type 2 micronutrients include zinc, potassium, and magnesium [1].

Growth deficits are further divided into the overlapping subcategories of wasting, stunting, and underweight [5]. Being wasted is strictly defined as a weight-for-height *z*-score (WHZ) below −2, stunted as a height-for-age *z*-score (HAZ) below −2, and underweight as a weight-for-age *z*-score (WAZ) below −2 [6]. Both being stunted and wasted overlap heavily with the category of children who are underweight [5]. Up to 93% of underweight children are also stunted or wasted and 17% accumulate a degree of both deficits [5].

All growth deficits are associated with an increased risk of mortality compared to children with no growth deficits. Moderately (*z*-score between −2 and −3) stunted, underweight, and wasted children have a 2.3-, 2.6-, and 3.4-fold increased risk of mortality, respectively [7]. Severe (*z*-score below −3) stunting, underweight, and wasting are associated with 5.5, 11.6, and 9.4 increased risk of mortality, respectively [7]. Accumulating several deficits increases substantially the mortality risk: children with both stunting and wasting, either moderate or severe, have a 12.3-fold increase in their risk of mortality [8].

Currently, only severe wasting is intentionally addressed via therapeutic feeding [9–11]. Children are admitted to therapeutic feeding programs for severe acute malnutrition (SAM) if their WHZ is below −3, they have a mid-upper arm circumference (MUAC) below 115 mm or in the presence of edema [12]. MUAC is used to detect being wasted as it reflects muscle mass deficits [13, 14], predicts mortality [15], is quick and easy to measure, and thus is used in community screening campaigns to identify malnourished children [9].

However, it is unclear whether the use of WHZ <−3 and MUAC <115 mm is optimal for capturing all children at the highest risk of mortality and for targeting all that would benefit from nutritional supplementation. An analysis of community-based cohort data from Senegal found that up to 62% of children at risk of death associated with growth deficits were missed when using WHZ <−3 and MUAC <115 mm [16].

Nutritional treatment is currently prescribed based on the weight of the severely wasted child and aims to fulfill 100% of the daily nutrient needs of the recovering child [10, 11]. However, recent evidence has shown that it is possible to reduce the dose of ready-to-use therapeutic food (RUTF) prescribed without undermining the recovery of treated children [17, 18] all the while making treatment less costly per child [19].

Beyond reducing the RUTF dose prescribed, simplifying the dosing regimen to 1 or 2 daily sachets per child based on the MUAC category instead of weight has been proposed and tested with encouraging results [17, 18]. The advantage of such a simplified dosage is both its simplicity to health agents prescribing treatment as well as to caregivers administering the treatment to children at home.

The simplified dosing regimen is only one aspect of a broader current initiative to towards simplifying malnutrition treatment [20]. Despite some improvements in treatment coverage over the past decade, still an estimated 70% of children with SAM and even more with moderate acute malnutrition (MAM) are left untreated in the community [21]. This is mostly attributed to the inexistence of screening and treatment services [22] which are often seen as burdensome and complex to run [23]. Simplified approaches to the detection and treatment of malnutrition have been proposed as a solution to scale up treatment services and enhance continuity of care [24].

One commonly proposed simplification is the combination of treatment of moderately and severely wasted children under the same program to ensure a continuity of care for wasted children and also prevent deterioration into more severe forms. Currently, children with MAM are eligible for supplementary feeding which can be in the form of a nutritional supplement providing 500 kcal/day [22]. However, treatment is oftentimes not available [22]. Recent recommendations on MAM treatment have also created some confusion about whether MAM should always be treated [25] despite evidence that moderately wasted children benefit from supplementation [26].

Moreover, there is growing interest in breaking the separation between stunting and wasting [27] and thus expanding the focus from treatment to include secondary prevention of severe malnutrition [28]. Traditionally, stunting has been largely seen as a non-treatable condition and is typically addressed through preventive interventions [29]. However, evidence is emerging that previous wasting episodes may be driving the development of stunting [30] and that stunting pre-disposes to wasting [31]. Both conditions seem to share some common risk factors and potential mechanisms driving the increased risk of mortality [27]. Particularly in contexts where both stunting and wasting are prevalent, addressing both seems critical to sustainably decrease the associated mortality risk.

Another simplification is the use of only MUAC and edema to admit children to treatment [32–36]. This enables leaving out the more time-consuming and error-prone procedures of weight and height measurements and the complex interpretation of WHZ tables. Admitting children with a MUAC <125 mm or edema to malnutrition treatment programs has been proposed as a means to facilitate decentralization of treatment to the less trained community health worker force [32, 34, 35].

However, using only MUAC <125mm and edema still leaves out children who are at risk of mortality associated with other growth deficits [16]. In a Senegalese cohort study that followed children in time, 34% of deaths among children with some growth deficit were not detected with a MUAC <125 mm [16]. On the other hand, all of these would have been captured with the use of WAZ <−2.8 [16]. This finding has led to the suggestion that WAZ <−3 could be added as an independent admissions criterion for therapeutic feeding programs in addition to MUAC <125 mm. This would enable capturing all wasted, stunted, and underweight children with an increased risk of mortality due to their growth deficits.

Currently, there is little evidence to inform the debate about whether children with MUAC ≥125 mm and WAZ <−3 respond to treatment and, if so, what treatment protocol should be used. There are numerous studies identifying optimal treatment protocols for children with MUAC <125 mm [32, 34, 35]. These protocols reduce the RUTF dose progressively towards the end of the treatment. Clinical trials have demonstrated that these alternative dosing protocols are non-inferior to the standard dosing regimen that keeps a “full” RUTF dose until the end of treatment [33, 34, 37]. However, to our knowledge, there is no data about optimal therapeutic feeding protocols for children with MUAC ≥125 mm and WAZ <−3. Currently, children with WAZ <−3 but MUAC ≥125 mm do not receive any treatment in areas implementing simplified protocols with MUAC-only-based admissions.

This study will assess whether children with WAZ <−3 but MUAC ≥125 mm benefit from therapeutic feeding and whether a simplified protocol is at least as effective as the more complicated weight-based standard protocol for this population. The publication of this study protocol aims to improve transparency and share information to support the development of similar studies that seek to study the optimization of the diagnostic criteria for malnutrition treatment and the therapeutic dosing of RUTF.

### Objectives {7}

The primary objective of this study is to assess whether therapeutic feeding with a simplified protocol results in 1) superior nutritional outcomes compared to no therapeutic feeding and 2) non-inferior nutritional outcomes compared to the WHZ and weight-based dosing regimen currently used in CMAM treatment, 2 months after diagnosis among children aged 6–59 months with MUAC ≥125 mm and WAZ <−3.

The secondary objective of this study is to assess whether therapeutic feeding with a simplified protocol results in (1) sustainably superior nutritional outcomes compared to no therapeutic feeding and (2) sustainably non-inferior nutritional status compared to the WHZ and weight-based dosing regimen currently used in CMAM treatment, 6 months after diagnosis among children aged 6–59 months with MUAC ≥125 mm and WAZ <−3.

Additional objectives include assessing whether the simplified protocol results in fewer children presenting negative outcomes compared to no treatment and non-inferior treatment outcomes compared to standard treatment.

### Trial design {8}

The study is a parallel group individually randomized controlled trial with three study arms allocated unevenly to test both a superiority and a non-inferiority hypothesis: 209 children will be recruited for the control arm (only involved in the superiority hypothesis testing) and 594 children in each intervention group.

## Methods: participants, interventions, and outcomes

### Study setting {9}

The study will be conducted in 11 health centers in the Nara health district of the Koulikoro region in Southern Mali. The district has a surface of 30,000 km^2^ and a population estimated at 351,517 inhabitants in 2021 of which 20% are children below 5 years of age [38]. The region is predominantly arid land [39] and the population is subsisting on small-scale farming and herding [40]. The region is less affected by insecurity and conflict compared to the North and Center regions. Food security in the region has been relatively stable since 2018 and categorized as minimally insecure or stressed [41–44]. However, access to health care remains an issue with 29% of the population in the district living over 15 km away from basic services [45]. In the region, only 48% of children 12–24 months of age have been fully vaccinated and malaria prevalence is 22% among children under 5 years of age [46]. Acute malnutrition prevalence as measured by a WHZ <−2 was estimated at 10.9% in 2021 [47]. IRC has been present in the area since 2015 supporting the nutrition activities including strengthening community screening activities and health care staff skills in managing acutely malnourished children. At the start of 2022, the district counted 39 public health centers providing outpatient care and one inpatient facility. The criteria used to select the study sites out of the 39 existing health centers were related to accessibility for study teams (no acute security threat) and for study population (access maintained between villages and health center during rainy season), and sufficient population size covered by health center to enable a constant enrolment rate of participants. The list of the study sites can be found at the trial registration page at ClinicalTrials.gov: NCT05248516.

Since 2018, the district has been implementing a simplified protocol to treat acute malnutrition [32] admitting children based on their MUAC measure (<125 mm) or presence of bilateral edema and discharging children after 2 consecutive measures of MUAC ≥125 mm. The prevalence of MUAC <125 mm in children 6–59 months of age is estimated at 2.6% in the Koulikoro region according to the latest national nutrition survey in 2021 [47]. Based on the same data, the proportion of children with MUAC ≥125 mm but with WAZ <−3 is estimated at 2.7%.

### Eligibility criteria {10}

The study population will be composed of children living in the catchment area of 11 health centers of the Nara district where the study will be taking place. Inclusion criteria include (a) age between 6 and 59 months, (b) MUAC ≥125 mm, (c) WAZ <−3, (d) living in the study catchment area, and (e) expects to be able to continue follow-up visits for next 6 months. Children will not be included in the trial if they have (a) nutritional edema, (b) known peanut or milk allergy, (c) severe illnesses requiring inpatient level treatment (according to CMAM guidelines), (d) medical condition affecting food intake (lip and palate cleft, handicap etc.), and (e) has already taken part in the study.

### Who will take informed consent? {26a}

Eligibility for the study will be assessed at the research site by the research staff who are not routine health staff but are employed specifically to conduct the study. Once a child has been screened eligible at the research site, their caregiver will be informed about the study in the local languages by one of the 5 research personnel present at the study site speaking the caregivers’ language. Each research team is composed of a supervisor, 2 nurses, and 2 polyvalent workers. Once the caregiver has been given time to ask questions, they will be invited to participate, and if they consent, asked to sign or thumbprint the consent form.

### Additional consent provisions for collection and use of participant data and biological specimens {26b}

Participants will be asked for their consent to make de-identified data publicly available and use it for further research purposes.

## Interventions

### Explanation for the choice of comparators {6b}

The simplified treatment will be compared both to the current WHO-recommended standard treatment as well as to no treatment. The standard protocol is considered as the reference practice in the field and is needed as a comparator in order to judge whether the simplified protocol results in no worse outcomes compared to the standard treatment. The control arm is needed to establish whether simplified or any treatment actually results in improvement in nutritional status both in the short and in the long term.

### Intervention description {11a}

Intervention will start on the same day as children are confirmed eligible and caregivers have given their consent for participation. Children in the control group will not receive any nutritional treatment nor any other equivalent value non-food item. Children randomized to the simplified arm will all receive a nutritional treatment until being discharged. This nutritional treatment will consist of 1 sachet of RUTF per day for a maximum of 12 weeks or until reaching a WAZ ≥−3 for 2 consecutive visits. Children randomized to the standard arm will be categorized into 3 sub-groups: (1) those with a WHZ <−3, (2) those with a WHZ between −3 and <−2, and (3) those with WHZ ≥ −2. Children in the first sub-group will receive a weight-based RUTF dose that aims at providing 200 kcal/kg/day. The second sub-group will receive 1 sachet of RUTF per day. Both first and second sub-groups will be treated until WHZ ≥ −2 for 2 consecutive visits or for a maximum of 12 weeks. The third sub-group will receive no treatment. All children irrespective of their arm or sub-group will be followed up fortnightly until 12 weeks and then monthly until 24 weeks post-enrolment.

Exit criteria from treatment ire followed in the 2 intervention arms: standard and simplified treatment arms. Children in the control arm will not have exit criteria as they do not receive treatment and are only followed up.

Six exit categories exist from treatment: (1) recovery; (2) defaulting defined as missing 2 consecutive appointments at the treatment facility; (3) non-response, defined as not reaching the recovery criteria within 12 weeks; (4) died, defined as those cases of children that die during the trial, regardless of the reason of death; (5) transfer to inpatient care, defined as those cases that develop medical complications, as per the national protocol, and require inpatient treatment during the trial; (6) developing a MUAC <125 mm or edema.

Recovery is defined differently for children in the simplified and standard arm. In the simplified arm, recovery is defined as reaching a WAZ ≥−3 for 2 consecutive visits. In the standard arm, recovery is defined as reaching a WHZ ≥−2 for 2 consecutive visits. See Table 1 for the differences between arms.

**Table 1:** Differences in intervention and end of intervention criteria between study arms

PROCEDURES	Control arm	Simplified treatment arm	Standard treatment arm
Treatment provided	None	1 sachet of RUTF/day	Depending on the WHZ score: • WHZ <−3: 200 kcal of RUTF/kg/day • WHZ between −3 and −2: 1 sachet of RUTF/day • WHZ ≥−2: no treatment
Recovery criteria from treatment	None	WAZ >−3 on 2 consecutive visits	WHZ ≥−2 on 2 consecutive visits

All other exit categories except for MUAC <125 mm or edema or died continue in the study unless the caregiver is not willing to do so. All children requiring inpatient-level care will be referred to the health center’s head nurse or doctor for them to prepare a formal referral of the patient to inpatient care.

Children will be classified in 5 different categories at the exit from the study: (1) finished 6-month follow-up, (2) developed MUAC <125 mm or edema during follow-up and was referred to routine treatment, (3) abandoned study, (4) lost-to-follow-up, (5) died. Abandoned will be defined as those who do not come back to study visits but who have been contacted to confirm the child is still alive. Lost-to-follow-up will be defined as those who do not come back to study visits and who have not been reached to confirm whether the child is still alive.

### Criteria for discontinuing or modifying allocated interventions {11b}

There will be no criteria for changing treatment once allocated to a treatment arm. However, participants can withdraw their consent to participate in the study at any moment. Children developing medical complications will be referred to inpatient care where study-independent medical and nutritional treatment may be administered.

### Strategies to improve adherence to interventions {11c}

Upon RUTF distribution, caregivers will be reminded that the product is only intended to be given to the participating child and not to anyone else and advised on the correct daily dosing of the product. Consumption of RUTF will be monitored through questions on intake and by asking caregivers to return empty sachets at each visit.

### Relevant concomitant care permitted or prohibited during the trial {11d}

Any illnesses identified during study visits or outside of study visits in routine health care will be treated as per the Malian national protocol for the treatment of childhood illnesses. No prohibitions for concomitant care are applied. Children developing a MUAC <125mm or edema will be discharged from the study and referred to routine simplified treatment available at all study sites and throughout the district.

### Provisions for post-trial care {30}

Post-trial care can be sought via the routine health care available throughout the district. Any unexpected health problems that can be ascribed to participation in the study will be covered through a patient insurance which IRC has secured specifically for this trial.

### Outcomes {12}

Both primary and secondary outcomes will be reported for all 3 arms at both 2 (primary endpoint) and 6 months (secondary endpoint) post-enrolment. The primary outcome is the mean WAZ of children. WAZ has been shown to be strongly associated with mortality and thus acts as a proxy of the ultimate goal of reducing child mortality. Increase in WAZ can be both related to the increase in HAZ or WHZ, which is an added value as it enables appreciating and aggregating both outcomes that are positive. As a continuous measure, WAZ is well adapted to capture the response to treatment among the study population as it will more easily capture smaller benefits of treatment compared to categorical outcomes.

Secondary outcomes include (a) proportion of children with WAZ <−3, (b) mean MUAC of children, (c) mean WHZ and HAZ, (d) proportion of children with WHZ <−3 or HAZ <−3, (e) mean skinfold thickness measures and mean skinfold thickness per age percentile, (f) mean fat-free mass and fat mass, fat-free mass index and fat mass index, (g) mean hemoglobin measure, and (h) change in all these outcomes from enrolment to endpoint. In addition, the proportion of children exiting the study at any time point due to a MUAC <125 mm or edema or diseased or hospitalized will be reported across arms. Finally, treatment outcomes will be reported for the 2 treatment groups including the percent recovered, defaulted, non-response, transferred to inpatient care, and died. The study exit status will be reported for all 3 arms including completion of the 6-month follow-up, developed a MUAC <125mm or edema during the study, abandoned study, lost-to-follow-up, and died.

### Participant timeline {13}

**Fig. 1:**
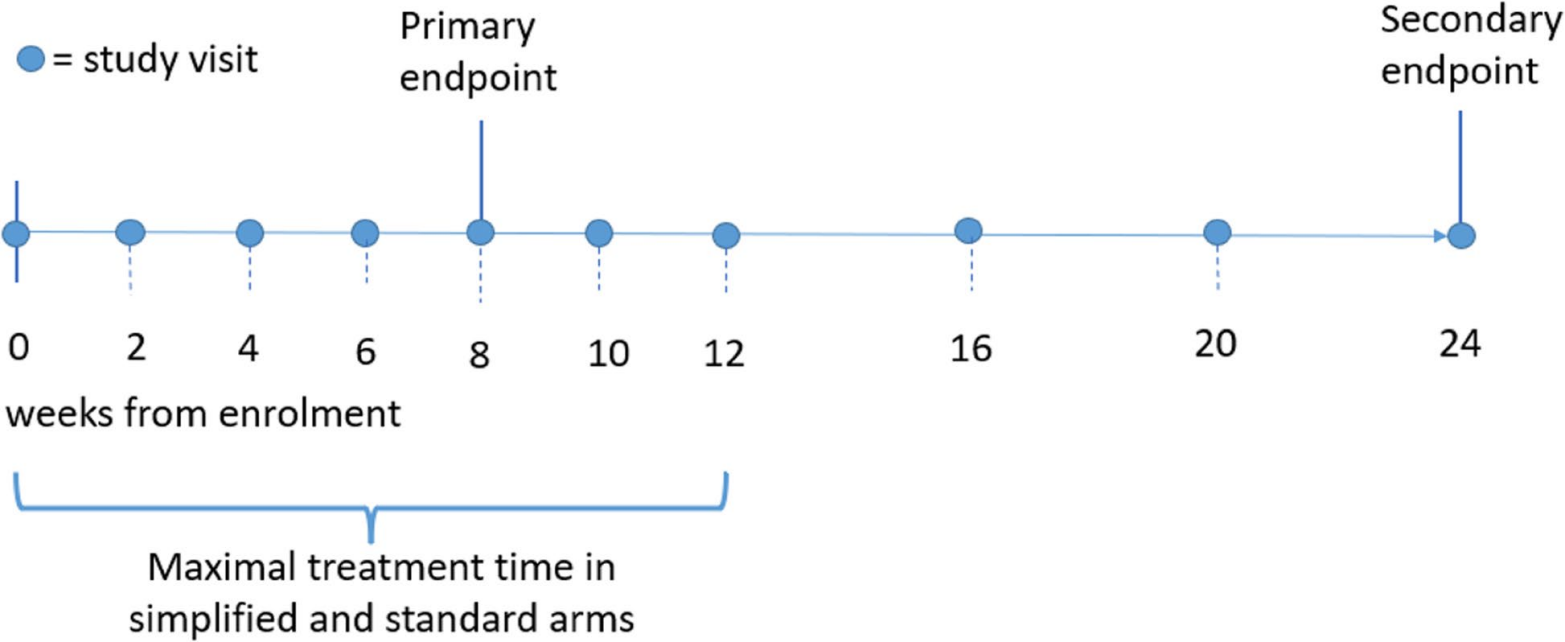
Timeline of study visits for all participants and intervention for participants in the simplified and standard arms

Study participants will be followed up fortnightly for the first 12 weeks and then monthly until 6 months. Figure 1 illustrates the visit schedule for any study participant and Table 2 presents the timing and frequency of different study procedures.

**Table 2:** Timing and frequency of different study procedures for children enrolled in the ComPAS low-WAZ RCT in Mali

Procedure	Week prior to enrolment	Enrolment	At each fortnightly and monthly visit	At 2 months and 6 months
Pre-screening for eligibility	X			
Eligibility		X		
Consent		X		
Socio-economic questionnaire		X		
Anthropometry (weight, height, MUAC)		X	X	X
Clinical exam		X	X	X
Body composition		X		X
Hemoglobin		X		X

### Sample size {14}

Based on previous work on a similar population and similar intervention [48] we assumed a similar response to treatment and a similar SD for WAZ. Thus, for looking at hypothesis 1, we set an objective of measuring a meaningful impact of the simplified treatment (compared to no treatment) at a difference of 0.4 *z*-scores in WAZ. For calculating the sample size, we applied (a) SD of 1.1 WAZ, (b) power of 80%, (c) probability of type 1 error of 2.5%, and (d) obtained a sample size of 146 per group. Accounting for not having measures for 30% of children (particularly in the control arm) at 2 months due to (a) defaulting, lost-to-follow-up, or death (10%) or (b) development of MUAC <125 mm or edema (20%), we estimated needing 209 children in both control and simplified treatment arms each.

For looking at hypothesis 2 we set an objective of measuring a meaningful non-inferiority margin of 0.2 *z*-scores in WAZ. For calculating the sample size, we applied (1) SD of 1.1 WAZ, (2) non-inferiority margin of 0.2, (3) power of 80%, and (4) probability of type 1 error of 2.5% and obtained a sample size of 475 per group. Accounting for not having measures for 20% of children at 2 months due to (a) defaulting, lost-to-follow-up, or death (10%) or (b) development of MUAC <125 mm or edema (10%), we need 594 children in simplified and standard treatment arms each.

To be able to explore both hypotheses, we need 209 children in the control arm and 594 children in both the simplified and standard treatment arms meaning a total of 1397 children.

### Recruitment {15}

In the health areas where the study is taking place, study staff will be deployed to pre-screen children in the villages. The pre-screeners will move door to door and seek to identify children that are in the age range of 6–59 months with a MUAC >125 mm but WAZ <−3. Pre-screeners will be equipped with a calendar that helps the estimation of age as well as an electronic weight scale and tablet that calculates the exact WAZ score of the child. Once a child has been screened eligible by the pre-screener, they will receive a screening card and instructions to visit the IRC research team at the closest health center on a given day when research activities are taking place. Eligibility will be confirmed at the research site prior to proceeding to consent and participation.

## Assignment of interventions: allocation

### Sequence generation {16a}

The random sequences will be generated in STATA by one of the data managers. Stratified, blocked randomization will be used. Stratification will be done by recruitment site, i.e., separate random sequences will be generated for each of the eleven sites. Blocked randomization will be used, with varying block sizes.

### Concealment mechanism {16b}

Treatment allocation will be blinded to the investigators until data cleaning and initial data analyses have been completed. The main data file will contain an intervention code, which will need to be unblinded to know which code corresponds to which group. The intervention code will be kept in a separate file, only available to the person responsible for the provision of randomization lists. Once data collection and data cleaning are complete, the intervention code will be released to the investigators.

### Implementation {16c}

The random sequences will be generated by one of the data managers. Enrolment will be done by study staff present at the research sites. Participants will be given consecutive health center-specific study ID numbers that will determine to which study arm they belong to, according to the random sequences. The person giving the study ID will not have access to the randomization list. Thus, the randomization arm of the newly enrolled child will only be revealed to the study team and to the caregiver when the child has been enrolled.

## Assignment of interventions: blinding

### Who will be blinded {17a}

This is an open trial since participants cannot be blinded with respect to the intervention they are receiving. Study staff will also not be blinded. However, treatment allocation will be blinded to the investigators until data cleaning and initial data analyses have been completed.

## Data collection and management

### Plans for assessment and collection of outcomes {18a}

#### Socio-economic questions

A socio-economic questionnaire will be administered upon enrolment to the study. Information will be collected through an individual interview with the caregiver. The questions include socio-economic indicators, family size, and main income sources of the household.

#### Anthropometry

Weight, height, MUAC, and edema will be measured in all children at every visit. All measures will be performed and recorded twice by alternating the roles of the measurer and the assistant, and the mean used for analysis. Weight will be measured to the nearest 0.1 kg with an electronic scale (SECA scale). Length and height will be measured to the nearest 0.1 cm with a wooden height board with graduated index strips in millimeters (UNICEF model). MUAC will be measured with a non-stretchable white MUAC tape on the left arm to the nearest millimeter (mm).

#### Body composition

Body composition will be measured both by measuring skinfold thickness as well as the bioelectrical impedance. Body composition will be measured at enrolment and at 8 and 24 weeks from enrolment.

Skinfold thickness will be measured with the GIMA code 27346 device that gives an electronic reading of the result with 1mm precision. Skinfold thickness measures will be performed at the sub-scapular and triceps levels from the right side of the child with the child in a sitting position. Repeated measures will be performed for each site altering the measurer and the assistant. Bioelectrical impedance will be analyzed with the BodyStat 500 device that provides measurements for the resistance, reactance, and phase angle. A repeat measurement will be performed.

#### Hemoglobin

Hemoglobin will be measured at enrolment and at 8 and 24 weeks from enrolment using a HemoCue 301 device and by applying a finger prick technique to obtain a drop of peripheral blood. Devices will be checked regularly against Eurotrol control solutions for low and normal levels.

#### Clinical exam

The study nurses will conduct a standard clinical consultation at each visit for all children irrespective of study arm. The clinical exam will include a history of recent illness and the clinical assessment of the child (i.e., temperature, respiratory rate, pulse rate, cough, ear, and nasal discharges) according to the national protocol for the management of SAM [49]. Past and present symptoms, vital signs, diagnosis, and treatments prescribed will be recorded. The clinical assessment will also serve to monitor the development of complications that need inpatient care. All serious adverse events or development of complications will be immediately reported to the study supervisor and the child referred to inpatient treatment if needed. Adverse events include morbidities. Serious adverse events include death, development of any grade of edema, development of a MUAC <125mm, and development of medical complications requiring inpatient care.

Complications are based on the national CMAM referral criteria [49].

#### Training

Study staff will be trained on all study procedures specific to their role during a 2-week-long theoretical and practical training. A standardization exercise as per the ENA SMART survey will be performed prior to the launch of the study to ensure the capacity of the polyvalent agents to accurately and precisely perform all anthropometric and body composition measurements. In case of non-conform results, re-training will be arranged and a new standardization organized until results return with acceptable accuracy and precision. Periodic anthropometric standardization exercises will be performed during the trial.

### Plans to promote participant retention and complete follow-up {18b}

In recognition of the efforts caregivers make when participating to the study, they will receive a small compensation of 1000 cfa at each visit aimed at offsetting the opportunity and travel cost of participation.

Participants will be encouraged to attend all study visits and reminders will be done through telephone and home visits by community focal points. Continuation of study visits until 6 months post-enrolment will be encouraged regardless of treatment outcomes (defaulting, non-response, etc.).

### Data management {19}

All questionnaire data and measurements will be recorded through digital data collection using tablets and the software CommCare (https://www.dimagi.com/commcare/). A unique barcode with participant ID will be assigned to each child and used to identify the child at each visit. Data collected on the tablets is transferred automatically to the data base on the server and aggregated to the existing patient record and data base. Data will be treated with the help of automated error identification within CommCare and through Power BI to check the coherence, consistency, and completeness. Regular supervision and refresher training workshops will be organized to maintain a high data quality.

Data will be reviewed weekly to monitor the data quality and confirm a good balance in the allocation of children in the 3 study arms. Baseline data monitoring will include reporting the baseline anthropometric characteristics of children by arm, their age and sex distribution, and some basic socioeconomic variables. Outcome data quality monitoring will include looking into anthropometric measurements and checking the normality of their distributions, precision in terms of an even use of decimals and mean differences between 2 subsequent measurements, and looking for any unusual increments or reductions between measurements from subsequent visits. The quality of the intervention will be monitored by checking the concordance between the amount of RUTF received as specified by the study arm and the child's anthropometric measures.

### Confidentiality {27}

Personal identifiers, including names of participants and their date of birth, will be collected on paper registries and on the electronic data forms. Paper registries will be kept with the study staff at all times and in locked boxes when not in use. Personal identifiers are kept in the electronic data base as long as the study is running, to be able to cross-validate participant identity. After data cleaning and verification, personal identifiers will be removed from the data files. Photos and videos of participants will not be taken without caregiver’s specific consent (Supplementary file 2).

### Plans for collection, laboratory evaluation and storage of biological specimens for genetic or molecular analysis in this trial/future use {33}

No biological specimens will be collected in this trial. Hemoglobin and malaria rapid tests will be conducted using the finger prick method and extracting a drop of blood from the participant directly to the single-use microcuvettes for analysis. The results of the tests will be noted down and the biological waste including the finger prick device and the microcuvette disposed of immediately.

## Statistical methods

### Statistical methods for primary and secondary outcomes {20a}

In reporting the trial outcomes, we will follow the CONSORT 2010 statement for reporting parallel group randomized trials [50]. This will include reporting participant flow through the trial as per the CONSORT guidance [50]. All statistical analysis will be done using STATA® software or similar. Baseline characteristics will be reported by the study arm. Linear mixed models will be fitted to analyze the primary outcome and all continuous outcomes while logistic mixed-effects models will be fitted for binary or categorical outcomes. Random effects will include participants, study sites, and study teams. Adjustment for block randomization will be applied by means of random effects. Adjustment for confounders such as age and sex and outcome at enrolment will be included. To investigate effect modification, two-way interactions between intervention group and age group, sex, and degree of undernutrition (WHZ, WAZ and HAZ categories) will be assessed.

Anthropometric outcomes at 2 and 6 months post enrolment will be analyzed by adjusting for the exact number of days from enrolment to the measurement date. In addition, the treatment outcomes will be described for the 2 treatment groups, and the study exit status for all 3 study groups using count and percentage.

### Interim analyses {21b}

A data safety monitoring board (DSMB), comprising of independent members with experience in nutrition epidemiology and humanitarian nutrition, will review the outcomes and adverse events at mid-point of the trial.

### Methods for additional analyses (e.g., subgroup analyses) {20b}

Sub-group analyses will include children with (1) concurrent wasting (WHZ <−2) and stunting (HAZ <−2) versus only wasted versus only stunted, (2) severe wasting (WHZ<−3) versus moderate wasting (WHZ ≥−3 and −2) and no wasting (WHZ >−2) and (3) severe stunting (HAZ <−3) versus moderate stunting (HAZ ≥−3 and −2) and no stunting (WHZ >−2), at enrolment to study.

### Methods in analysis to handle protocol non-adherence and any statistical methods to handle missing data {20c}

All analyses will be carried out in an all available case basis, i.e., all study participants will be included in the analysis. In case of missing outcome data at the primary and secondary endpoints of 2 and 6 months respectively, imputation will be performed using key variables such as age, sex, time since enrolment, randomization arm, treatment status, other anthropometry, and any other (previous or later) outcome measure data points. The primary analysis will apply a “de facto” principle [51] answering to the question of whether the treatment works in a real-life setting, and will thus analyze the data irrespective of degree of adherence to their treatment group. In addition, a “de jure” analysis will be performed to estimate the efficacy of the treatment in a best-case scenario, adjusting for the actual dose obtained, simulating a per-protocol analysis, as per the recommendations for non-inferiority analysis [52, 53]

### Plans to give access to the full protocol, participant-level data, and statistical code {31c}

The last version of the full study protocol and standard operating procedures will be published with the article on the main outcomes of the trial. Deidentified participant-level data will be made publicly available on Zenodo once primary analyses are ready. The statistical code for all analyses reported in peer-reviewed articles will be included as supplementary files of the article.

## Oversight and monitoring

### Composition of the coordinating center and trial steering committee {5d}

This trial has been designed and set up in collaboration between the International Rescue Committee, University of Sciences, Technics and Technologies of Bamako (USTTB), and the Ministry of Health and Social Development (MoHSD) of Mali. The co-investigator group is composed of 2 researchers from the IRC, 1 researcher from USTTB, and 1 nutritionist from the MoHSD of Mali. This group will be meeting at least monthly during the trial to review trial progress and discuss any implementation issues and needs for revisions to protocol or implementation procedures.

The day-to-day study activities will be managed by a full-time study manager based in Nara, supported by a deputy manager. Four field teams will be performing the actual study intervention and data collection, and a total of 8 pre-screeners will be deployed in the community to screen children based on the eligibility criteria. Each field team will be composed of 5 members including 2 polyvalent agents in charge of the consent process, anthropometric measurements, questionnaires, and body composition measurements, 2 study nurses who will perform clinical checks, hemoglobin measurements, and prescribe and administer any medication and 1 supervisor that will supervise the team, administer the nutritional treatment according to the study arm and compensate participants at each visit. The full field team will be meeting monthly to discuss any implementation issues and be re-trained on processes as needed.

Data will be managed by 2 data managers who will be reviewing data quality and progress of implementation on a weekly basis. A list of data points for double-checking will be shared weekly with the field team in order to flag data points that seem unusual and where cross-checking of electronic data is needed against any paper forms. These flags can for example be unusually high increases in weight between 2 consecutive measurements, or any decrease in height over time. A data review meeting will be held fortnightly involving the data managers, the study manager, and 2 investigators to alert the team on any specific issues and decide on actions (training or other). The data reviews will include checking the baseline balance between randomization groups, quality of outcome data collected and completeness of data collected, and quality of intervention. Only baseline data will be stratified per arm to monitor randomization success while outcome data will be kept separate from the randomization code to avoid pre-mature conclusions of the trial.

The trial is guided by a scientific committee of experts in pediatrics, humanitarian nutrition, epidemiology, and statistics from the London School of Hygiene and Tropical Medicine, University of Copenhagen, University of Tampere, Brixton Health, and National Institute of Public Health Denmark. This group will be receiving updates on the trial progress and will be involved in any decisions to revise protocol or implementation processes.

### Composition of the data monitoring committee, its role and reporting structure {21a}

A DSMB, comprising of independent members with experience in nutrition epidemiology and humanitarian nutrition, will review the outcomes and adverse events at mid-point of the trial. The committee is composed of a President and a Statistician. The president will be responsible for convening meetings at suitable intervals and communicating with the investigator group on the committee’s work and decisions. The statistician will be responsible for performing the interim analyses. Both members will decide together on the timing and data included in the interim analysis and will be involved in the discussion on the findings and recommendations to be made to the investigator group.

### Adverse event reporting and harms {22}

All serious adverse events will be immediately reported to the study supervisor and the child referred to inpatient treatment if needed. Serious adverse events include death, development of any grade of edema, development of a MUAC<125mm, and development of medical complications requiring inpatient care. In case of death, an investigation to the cause of death will be made within 48 h of the information and a report will be written and forwarded to the DSMB and IRBs within 72 h.

### Frequency and plans for auditing trial conduct {23}

Three study investigators, 1 from IRC, 1 from USTTB, and 1 from MoHSD Mali will be performing field supervision to monitor the respect of the study processes. A week-long field supervision is planned for every 3 months. In addition, the study manager and their assistant will perform regular (weekly) field supervision to monitor the respect of the ethical and other implementation processes.

### Plans for communicating important protocol amendments to relevant parties (e.g. trial participants, ethical committees) {25}

Any changes to the study protocol, unless the recommendation comes from the DSMB, will require ethical approval prior to implementation, both from the IRC IRB and the ethics committee of the USTTB. Changes will be communicated to the study Scientific Advisors through email and the study participants verbally by field team supervisors and to the community through community focal points.

### Dissemination plans {31a}

The investigators plan to communicate the study results at the community, district, national, regional, and global level through presentations and discussions with relevant groups the investigator group are engaged with. At the community level, this will include holding debriefings of the project with the community focal points (*n*=200) that will be in charge of transmitting the messages to the study participants and communities. At the district level, this includes the Head Physician of the District, the District Health Lead team, and the Heads of the participating health centers and their nutrition focal points. At the national level, these include the Malian Nutrition Technical Group which involves all national nutrition actors including the MoHSD Sub-Division of Nutrition, UNICEF, WFP, and implementing non-governmental organizations (NGOs). Results will also be discussed with the Malian MoHSD Sub-Division of Nutrition to draw locally adapted recommendations. At the regional level dissemination activities will include a presentation and a discussion with the West- and Central-Africa Regional Nutrition Working Group sub-group on wasting which is headed by UNICEF. At the global level dissemination activities will include presentations and discussions with the Global Nutrition Technical Alliance, the Wasting Stunting Technical Interest Group which includes prominent nutrition researchers working in this sphere, and the Simplified Approaches Working Group.

## Discussion

This individually randomized controlled 3-arm trial evaluates the response to treatment of children with WAZ <−3 but MUAC ≥125 mm when treated with a simplified protocol versus standard protocol versus no treatment. If the results show that treatment is superior to no-treatment then this speaks for adding WAZ as an additional and independent admission criteria to MUAC <125 mm to treatment. If this study shows that the simplified treatment is non-inferior to standard treatment, then this contributes to the growing evidence base that the simplified protocol is as effective as the standard protocol in correcting childhood malnutrition.

## Trial status

Recruitment of trial participants began on the 29th of August 2022 and was completed in October 2023. Follow-up of the last patients should then be finished by the end of April 2024, at which point data will start being analyzed. This protocol is version number 3.2 dating 15 November 2022.

### Supplementary information

**Additional file 1.**

**Additional file 2.**

**Additional file 3.**

## Data Availability

All study investigators will have access to the final dataset. De-identified data of the trial will be made publically available once main analyses have been performed and manuscripts accepted for publication. No biological specimens will be collected or stored.

## Abbreviations

CSB: Corn-soy blend
DSMB: Data safety monitoring board
ENSA: Enquête nationale sur la sécurité alimentaire et nutritionnelle
HAZ: Height-for-age *z*-score
IRB: Institutional Review Board
IRC: International Rescue Committee
LNS: Lipid-based nutrient supplement
MoHSD: Ministry of Health and Social Development
MUAC: Mid-upper-arm circumference
RUTF: Ready-to-use therapeutic food
SMART: Standardized monitoring and assessment of relief and transitions
UNICEF: United Nations Childrens’ Fund CSB
USTTB: University of Sciences, Technics and Technologies of Bamako
WAZ: Weight-for-age *z*-score
WFP: World Food Program
WHZ: Weight-for-height *z*-score
