# Supplementary material for: Nutritional treatment of children 6–59 months with severely low weight-for-age z-score: a study protocol for a 3-arm randomized controlled trial
Source: Trials. 2024 Jan 8;25:30. doi: 10.1186/s13063-023-07890-0 (PMC10773065; doi:10.1186/s13063-023-07890-0)
Supplement: Supplementary file 1 — Additional file 1. [file 13063_2023_7890_MOESM1_ESM.pdf]

CONSENT FORM FOR WAZ-STUDY

	YES	NO
Staff explained the study to me in a language I understand. I had time to think about this information. I was able to ask questions. All of my questions have been answered.	☐	☐
I understand that it is my choice to join this study or not. I understand that I can stop participating in this study at any time, for any reason. If I stop participating, nothing bad will happen. My child will still receive health services. I do not give up any legal rights by participating in this study.	☐	☐
I understand that any personal information about me or my child, such as his/her name or address, will be kept private. Information that does not identify my child, such as the child's weight or height, may be used in other research studies. Information that cannot be linked to me or my child may be shared publicly.	☐	☐
**I am responsible for ____________________________. I accept that he/she will participate in this study.**	☐	☐
**I accept participating in this study.**	☐	☐
I accept you conduct the skinfold measure and the bioelectrical impedance analysis on my child	☐	☐
I accept you take a drop of blood from my child's finger	☐	☐

NAME AND SIGNATURE OF THE PARTICIPANT

________________________	________________________
(Name of the child)	(Study ID)

________________________	____________	____________________
(Name of the caregiver)	(Date)	(Signature or fingerprint)
________________________	____________	____________________
(Name of the impartial witness)	(Date)	(Signature or fingerprint)

NAME AND SIGNATURE OF THE RESEARCH STAFF

______________________	____________	____________________
(Name of the IRC staff)	(Date)	(Signature)
