# Supplementary material for: Nutritional treatment of children 6–59 months with severely low weight-for-age z-score: a study protocol for a 3-arm randomized controlled trial
Source: Trials. 2024 Jan 8;25:30. doi: 10.1186/s13063-023-07890-0 (PMC10773065; doi:10.1186/s13063-023-07890-0)
Supplement: Supplementary file 2 — Additional file 2. [file 13063_2023_7890_MOESM2_ESM.pdf]

Invitation to Participate in a Research Study
WAZ-study

We would like to invite your child to join our research study. You can say yes or no. We would like to give you some information to help you decide. We will tell you why we are doing this study. We will also tell you what we will do as part of the study. We will answer any questions you may have. This may take about 20 minutes. Please feel free to talk to others about the study if you wish.

We will now tell you about the study and what will happen if you take part.

Please feel free to ask any questions. We are happy to assist you.

Who are we?

This study is led by the IRC, the Mali Ministry of Health Subdivision of Nutrition and the University of Sciences, Techniques and Technologies of Bamako. The project is funded by private donors.

What is this research about?

This study is trying to find the best treatment for children with a low weight for their age. We are doing this research to find out whether underweight children benefit from a treatment. We are also studying what amount of treatment is best. Currently in Nara there is only treatment available for children who have a small arm. But some children, for example older children may be small in size but not have small arms. These children may still benefit from treatment.

Who are invited to join?

Any child aged below 5 years but above 6 months who has a low weight for their age but whose arm circumference is green (show mid upper arm circumference tape) is eligible to participate. These children are underweight and may benefit from nutritional treatment. This is what the study is about to investigate. Currently those children are not treated in Nara. This study will take place in 11 health centers in Nara, Mali. A total of 1,500 children will be included in this study.

What will happen to my child if we participate?

Children who join the study will be assigned to one of 3 groups. The child’s group is chosen at random. The study staff cannot decide which group a child will join. The caregiver also cannot decide which group a child will join.

The first group of children will meet every month with health staff. This group will not receive plumpy’nut unless their arm measurement becomes yellow or red. Plumpy’Nut is this (show) red sachet that is given to children when they are malnourished. It helps children gain weight and become stronger. The second group will need to come to the health center weekly to receive one sachet of plumpy nut per day. Once their child has gained sufficient weight we will stop giving Plumpy’Nut and ask the mother to come to the health center monthly. The third group will receive different amounts of Plumpy’nut based on their weight and height. Children in this third group will receive between zero and three packs per day. Depending on how much Plumpy’nut they receive, they will need to come every week or every month to the health center.

If you agree to participate in the study:

- Your child will be followed up for 6 months.
- You will bring your child to this site for follow-up on a fortnightly basis at first for 3 months and then on a monthly basis for the last 3 months. The study team will explain when you should come.
- An interviewer will ask you some questions about your household. There are no right or wrong answers. You can refuse to answer any question.
- Your child's height, arm and weight will be measured today and at every visit.
- Your child's body composition will be measured by folding the skin at the back and on the arm and by conducting a completely painless and safe electric current in the body.
- Your child's blood levels in iron will be measured by obtaining 1 drop of blood from the finger
- Your child will be examined by our study nurse today and at every visit. We will also ask questions about your child's health. Your responses will be written down on paper and in an electronic tablet (show them a tablet).
- This first visit will last approximately 1.5 hours. The following visits should not take more than 1 hour.

What are the benefits and risks for participation?

There are 2 ways your child may benefit from joining this study. First, your child will be examined regularly by a study nurse. If your child has any health problems, the nurse will treat your child or tell you where to get treatment. All treatment will be for free. Second, your child may also receive plumpy nut. This depends on which treatment group the study assigns to your child. Plumpy nut aims at helping your child gain weight.

There are also 4 risks with participation. First, while plumpy nut is meant to help your child gain weight, it can also cause diarrhea or vomiting. This happens not often but sometimes. Usually this passes in a couple of days. If the diarrhea or vomiting lasts for more than 3 days you should bring your child to the health center for a check up. Second, your child is likely to feel some pain when their finger is being pricked to obtain 1 drop of blood. We will make this measurement as quickly as possible and you also have the right to refuse this measure. Third, there is a risk of causing some discomfort for the child they move suddenly during the skinfold measure. To avoid this, we will instruct you how to hold the child so that they cannot move and thus they will not feel any pain when this measure is taken. Fourth, there could be a risk that some of the data that we are collecting from you gets into the hands of someone outside of the research team if for example the tablets or registries are stolen or get lost. We will do everything we can for this not to happen. The tablets are password protected and the paper registries are kept with the research team at all times.

Will there be any compensation for the participation?

Every time you come for a visit you will receive 1000 cfa to compensate you for your time and the cost of transportation. If you miss a visit and we have to come and visit you in your household you will not receive this compensation.

How will we use the information?

- The information collected will not be disclosed to anyone outside of the research team; it will remain private and confidential.
- The information collected will be stored in a locked file and in a database in a password protected computer.

- The information collected will be used for research purposes. If we make any information publicly available we will first take out all information that can make it possible to identify you or your child such as your or their name or address.
- Information that does not identify your child, such as the child's weight or height, may be used in other research studies.

Do you or your child have to participate in the study?

- Participation is entirely voluntary and optional. You are not obliged to take part in the study. You have been approached because you are a resident of the area where the study is being conducted and your child is underweight.
- You do not have to take part in the study even if others have agreed to.
- You can choose to stop participating in the study anytime without providing a reason. We will keep the information collected before you decide to stop.
- Your decision to participate or not, or to stop at any time, will not affect any services you may receive now or in the future
- You do not give up any legal rights by participating in this study.

Who do I contact if I have any questions or concerns during the study?

If you have any questions or concerns about any aspect of this study, please reach out to the research team listed below who will do their best to answer your questions.

Dr Koniba Diassana
Study Program Manager, IRC, Nara
+223 76 67 54 01
Address: bureau IRC de Nara, situé au quartier météo a 100m du poste de contrôle de l'axe Bamako

Dr. Moctar Tounkara
Study Investigator, USTTB, Faculty of Medicine, Bamako
+223 66 80 16 72

If you remain unhappy and wish to complain formally, or if you have any questions regarding your rights during this study, please contact the chairman of the Ethical Review Unit of University of Sciences, Technics and Technology of Bamako (USTTB). This unit has reviewed the study protocol and approved it. The contact details are:

Ethical Review Unit
University of Sciences and Technic and Technology of Bamako (USTTB), Faculty of Medicine, Pharmacy & Dentistry
Professeur Mamadou Marouf KEITA
President of the ethics committee
+223 66 72 20 22
+223 76 23 11 91

The IRC and the Faculty of Medicine hold insurance policies which apply to this study. If you experience harm or injury as a result of taking part in this study, you may be eligible to claim compensation without

having to prove that the IRC is at fault. This does not affect your legal rights to seek compensation.

If you are harmed due to someone's negligence, then you may have grounds for a legal action. Regardless of this, if you wish to complain, or have any concerns about any aspect of the way you have been treated during the course of this study then you should immediately inform the Study Coordinator:

Césaire Ouedraogo
Study Coordinator, IRC Bamako
+223 76 67 48 02

Who has reviewed this study?

All research involving human participants is looked at by an independent group of people, called a Research Ethics Committee, to protect your interests. This study has been approved by the Ethics Committee of the IRC in New York, and the Ethical Review Unit of the University of Sciences and Technic and Technology of Bamako (USTTB), Faculty of Medicine, Pharmacy & Dentistry.
